# Oral exposure to arsenic causes hearing loss in young people aged 12–29 years and in young mice

**DOI:** 10.1038/s41598-017-06096-0

**Published:** 2017-07-28

**Authors:** Xiang Li, Nobutaka Ohgami, Yasuhiro Omata, Ichiro Yajima, Machiko Iida, Reina Oshino, Shoko Ohnuma, Nazmul Ahsan, Anwarul Azim Akhand, Masashi Kato

**Affiliations:** 1 Department of Occupational and Environmental Health, Nagoya University Graduate School of Medicine, Nagoya, Japan; 2 Nutritional Health Science Research Center, Chubu University, 1200 Matsumoto, Kasugai, 487-8501 Aichi, Japan; 3 Voluntary Body for International Health Care in Universities, Nagoya, Japan; 4 Department of Genetic Engineering and Biotechnology, University of Dhaka, Dhaka, 1000 Bangladesh

## Abstract

There is no information on the association between oral exposure to arsenic (As) and hearing loss in humans or mice. In this combined epidemiological study and experimental study, the association of oral exposure to As with hearing loss in people aged 12–29 years and young mice was examined. Subjects in the exposure group (n = 48), who were drinking tube well water contaminated with As, showed significantly higher risks of hearing loss at 4 kHz [odds ratio (OR) = 7.60; 95% confidence interval (CI): 1.56, 57.88], 8 kHz (OR = 5.00; 95% CI: 1.48, 18.90) and 12 kHz (OR = 8.72; 95% CI: 2.09, 47.77) than did subjects in the control group (n = 29). We next performed an experiment in which young mice were exposed to As via drinking water at 22.5 mg/L, which is a much greater concentration than that in human studies. The exposure group showed hearing loss and accumulation of As in inner ears. *Ex vivo* exposure of the organ of Corti from mice exposed to As significantly decreased the number of auditory neurons and fibers. Thus, our combined study showed that oral exposure to As caused hearing loss in young people and young mice.

## Introduction

Contamination of arsenic (As) in drinking well water has been reported worldwide^1–3^. About 20 million people in Bangladesh are exposed to As by drinking well water in which As levels are above the national standard of 50 µg/L^4^. A previous study using univariate analysis showed that 10-year-old children in an As-polluted area had hearing losses at frequencies of 125, 250 and 8,000 Hz^5^. Thus, exposure to As is a potential risk for hearing loss in children. However, there has been no epidemiological study with multivariate analysis showing an association between oral exposure to As and hearing loss in young people aged 10–30 years.

Inner ears, which are important organs for hearing, contain the organ of Corti with inner hair cells (IHCs) and outer hair cells (OHCs) and spiral ganglion neurons (SGNs)^6^. A previous study showed that intraperitoneal injection of As at 200 mg/L into guniea pigs for 2 months resulted in morphological impairments of the inner ears^7^. Thus, it is possible that exposure to As induces hearing loss in experimental animals. However, there is no direct evidence about whether oral exposure to As via drinking water causes hearing loss in mice determined by auditory brainstem response (ABR). It is also unclear whether oral exposure to As via drinking water modulates the As level in inner ears of mice.

In this study, we examined the association of oral exposure to As with hearing loss in young people and young mice. For this purpose, we compared hearing levels of young people aged 12–29 years in a control group drinking tap water and an exposure group drinking tube well water contaminated with As in Bangladesh using a multivariate analysis adjusted for age, sex, body mass index (BMI) and smoking. We compared hearing levels by ABR and accumulation levels of As in inner ears of wild-type mice exposed to As via drinking water. We then tried to clarify the etiology of As-mediated hearing loss.

## Results

### Consumption of tube well water contaminated with As by people aged 12–29 years is significantly associated with hearing loss

**Figure 1:**
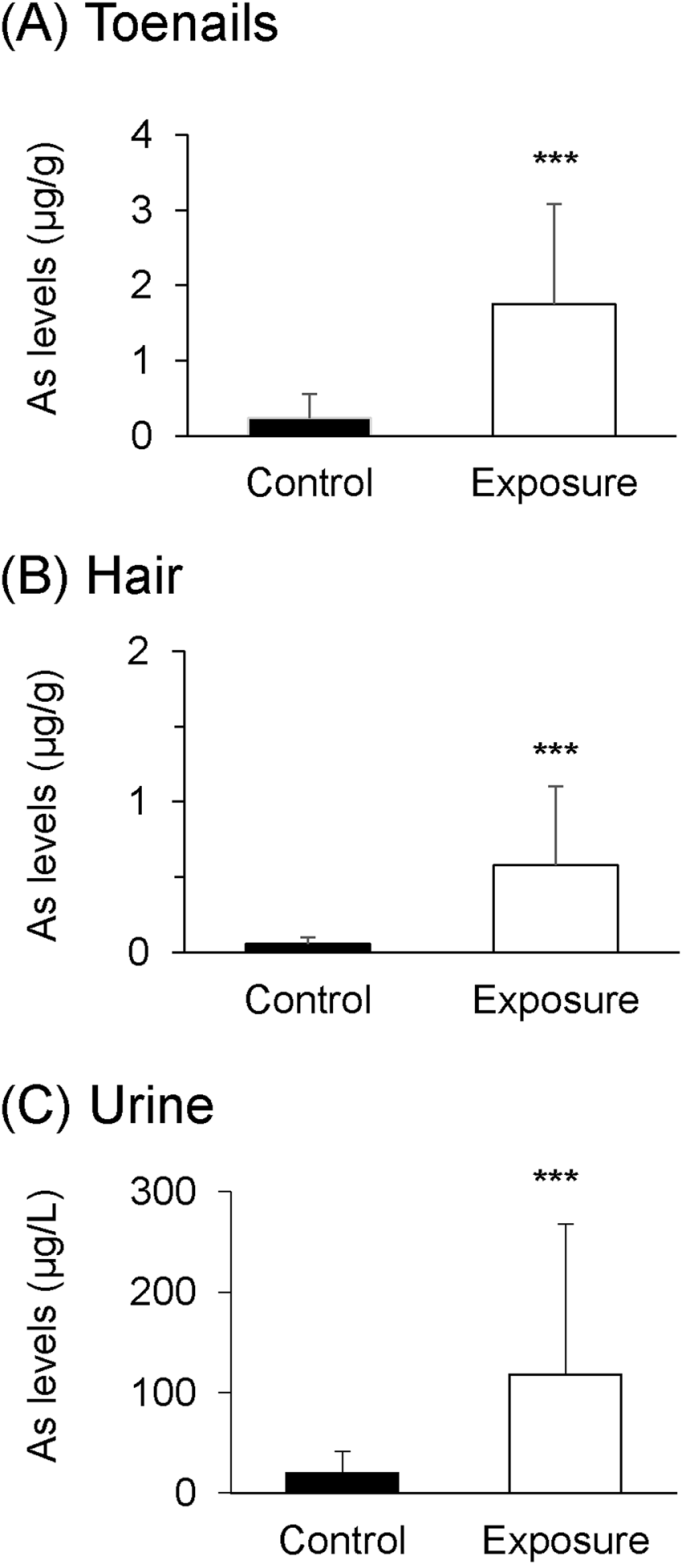
As levels in biological samples in from people aged 12–29 years drinking As-contaminated water and control group. As levels (means ± SD) in the control group (control; n = 29) and exposure group (exposure; n = 48) in toenails (**A**), hair (**B**) and urine (**C**) were measured. Significant differences (***p < 0.0001) were determined by the Mann-Whitney *U* test.

**Figure 2:**
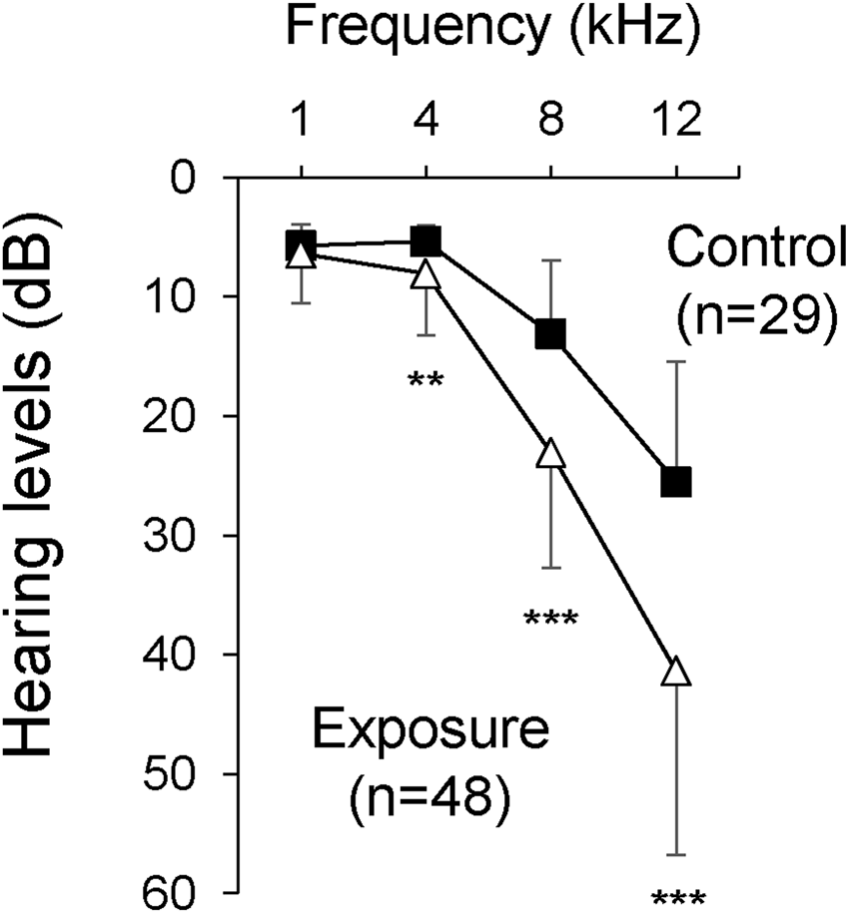
Hearing loss in people aged 12–29 years drinking As-contaminated water. Hearing thresholds (means ± SD) in the control group (control; n = 29) and the exposure group (exposure; n = 48) at 1, 4, 8 and 12 kHz were measured. Significant differences (**p < 0.01; ***p < 0.0001) were determined by the Mann-Whitney *U* test.

The basic characteristics of the subjects are shown in Table 1. The subjects were divided into a control group (n = 29) drinking tap water with 0.6 ± 0.7 µg/L of As and an exposure group (n = 48) drinking tube well water contaminated with As. Subjects in the exposure group shared four tube wells with As levels of 20.6, 53.8, 221.0 and 22.2 µg/L. The ages (mean ± SD) of subjects in the control and exposure groups were 21.52 ± 2.34 years and 20.38 ± 5.17 years, respectively. Levels of As in toenails, hair and urine in the exposure group were significantly higher than those in the control group (p < 0.0001) (Fig. 1). Hearing levels in the exposure group were significantly worse than those in the control group at 4 kHz (p = 0.0063), 8 kHz (p < 0.0001) and 12 kHz (p < 0.0001) (Fig. 2).

**Table 1:** Characteristics of the study participants (n = 77).

	Mean ± SD	Max	Min	Variables	Participants [n (%)]
Age (years)	20.81 ± 4.34	29	12		
Sex				Male	38 (49.35)
				Female	39 (50.65)
BMI				<18.5	15 (19.48)
	21.39 ± 3.69	31.87	13.78	18.5 ≤ BMI < 25	49 (63.64)
				≥25	13 (16.88)
Smoking history				No	72 (93.51)
				Yes	5 (6.49)
Drinking tube well water				Control	29 (37.66)
				Exposure	48 (62.34)

### Multivariate analysis of As-mediated hearing loss in people aged 12–29 years

We next performed binary logistic regression analysis for hearing loss with adjusted models for age, sex, smoking history and BMI^8–12^. We categorized hearing levels with the mean values (6 dB at 1 kHz, 7 dB at 4 kHz, 19 dB at 8 kHz and 35 dB at 12 kHz) of hearing thresholds in young people in this study. The multivariate analysis showed significantly higher risks of hearing loss at 4 kHz [odds ratio (OR) = 7.60; 95% confidence interval (CI): 1.56, 57.88], 8 kHz (OR = 5.00; 95% CI: 1.48, 18.90) and 12 kHz (OR = 8.72; 95% CI: 2.09, 47.77) in the exposure group than those in the control group (Table 2). To verify the validity of our model, we changed the cut-off values of the dependent variable dichotomizing hearing levels from 7 to 15 dB at 4 kHz, from 19 to 25 dB at 8 kHz and from 35 to 45 dB at 12 kHz. We found that significant correlations of hearing loss in the exposure group remained.

**Table 2:** Hearing loss in humans drinking tube well containing As.

	Adjusted OR and 95% Cls of hearing loss^a^			
	1 kHz (≥ 6 dB)	4 kHz (≥ 7 dB)	8 kHz (≥ 19 dB)	12 kHz (≥ 35 dB)
Control (n = 29)	Reference	Reference	Reference	Reference
Exposure (n = 48)	1.60 0.25–10.13	7.60* 1.56–57.88	5.00* 1.48–18.90	8.72** 2.09–47.77

Abbreviation: OR, odds ratio; CI, confidence interval. ^a^The adjusted variables were age^8^, sex^9^, smoking history^10, 11^ and BMI^12^, which were previously reported to affect hearing levels. *p < 0.05; **p < 0.01.

### Oral exposure to As caused accumulation of As in inner ears and hearing loss in young mice aged 3 months

**Figure 3:**
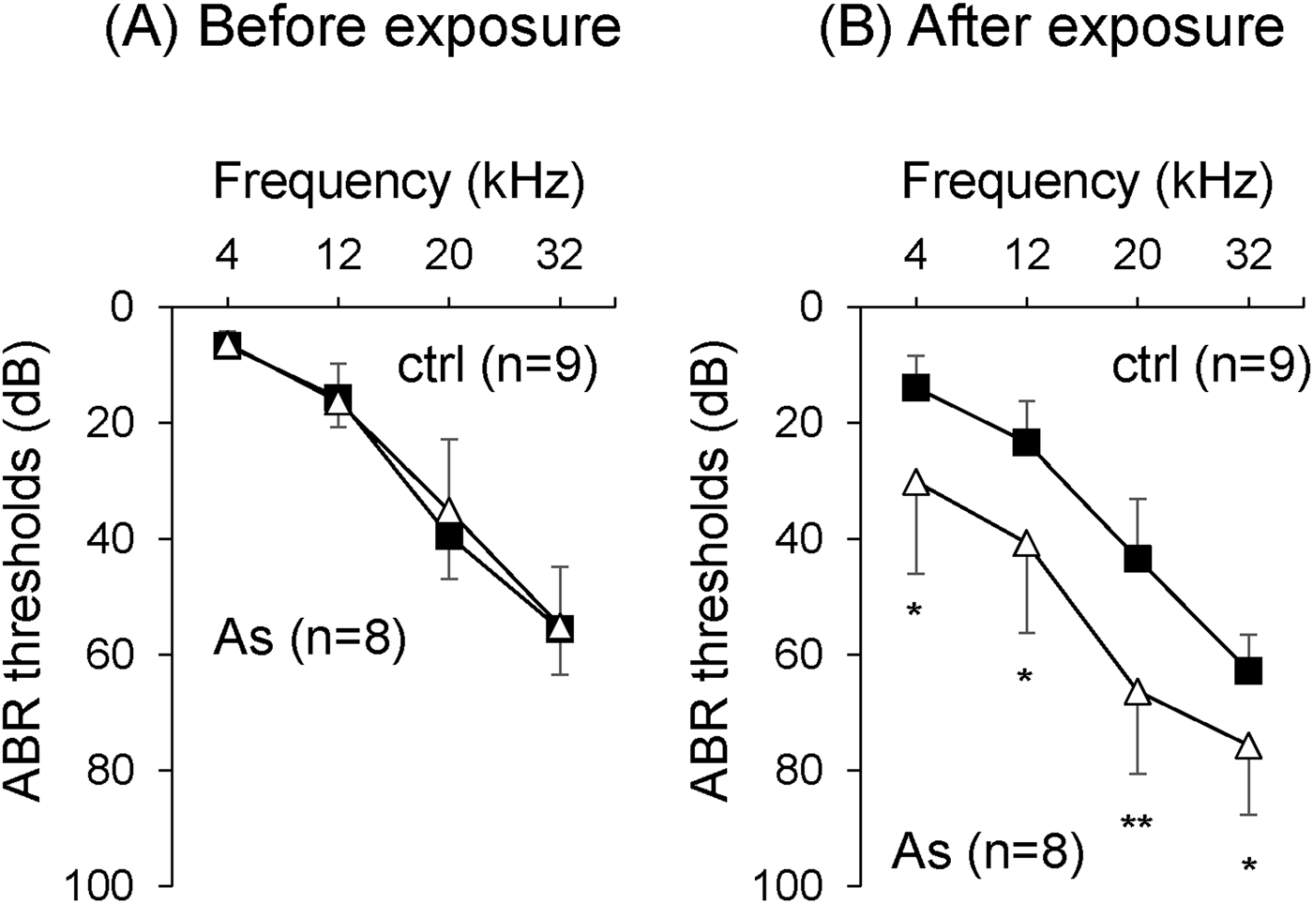
Oral exposure to As causes hearing loss in young mice aged 3 months. ABR thresholds (means ± SD) at 4, 12, 20 and 32 kHz in the control group (ctrl, closed squares, n = 9) and exposure group (As, open trianglers, n = 8) before (**A**) and after exposure to As (**B**) are presented. Significant differences (*p < 0.05; **p < 0.01) were determined by the Mann-Whitney *U* test.

**Figure 4:**
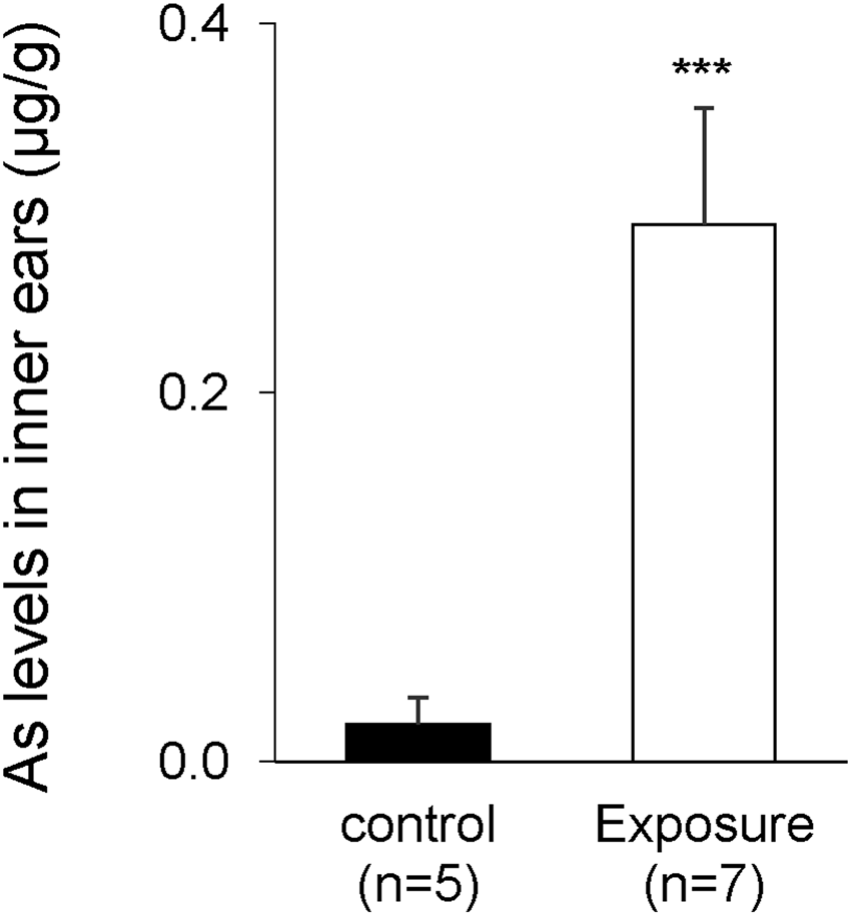
Accumulation of As in inner ears of young mice aged 3 months after oral exposure to As. As levels (means ± SD) in the control (closed bar, n = 5) and exposure (open bar, n = 7) group in inner ears are presented. Significant differences (***p < 0.0001) were determined by the Mann-Whitney *U* test.

**Figure 5:**
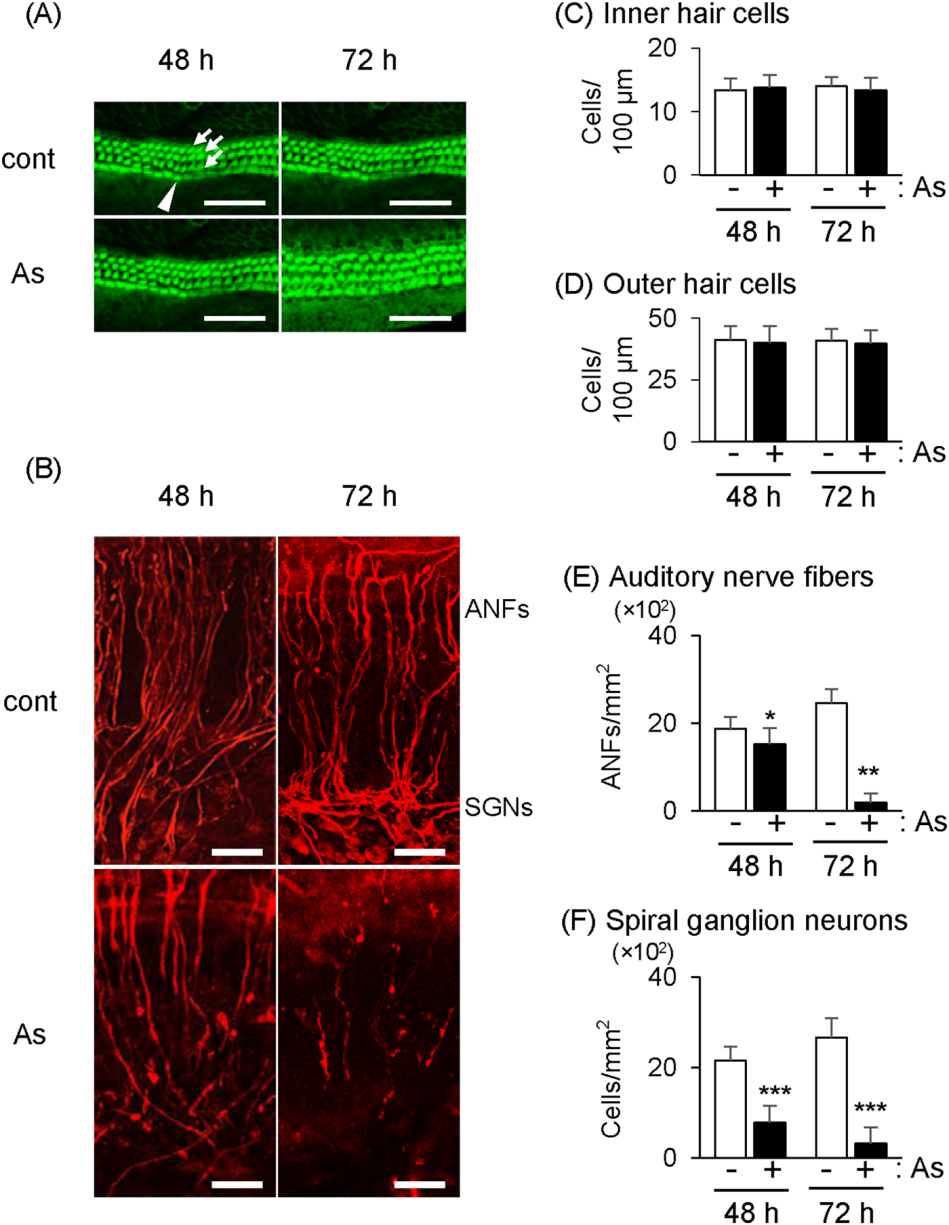
*Ex vivo* exposure of the organ of Corti to As at 0.3 µg/mL. (**A**,**B**) After *ex vivo* exposure of the organ of Corti to As at 0.3 µg/mL and no exposure (cont) for 48 and 72 hours, (**A**) inner hair cells (arrowhead) and outer hair cells (arrows) were stained with phalloidin and (**B**) spiral ganglion neurons (SGNs) and auditory neuron fibers (ANFs) were stained with anti-neurofilament 200 antibody. Scale bars: 50 µm. (**C**–**F**) Densities (means ± SD) of (**C**) inner hair cells, (**D**) outer hair cells, (**E**) auditory nerve fibers and (**F**) SGNs from the organ of Corti exposed to As (+, black bars, n = 10) and no exposure (−, white bars, n = 10) are presented. Significant difference (*p < 0.05, **p < 0.01, ***p < 0.0001) from the control was analyzed by the unpaired t-test.

Based on the results obtained by our epidemiological study, we next performed an experimental study to verify hearing loss in mice orally exposed to As at 22.5 mg/L via drinking water for 2 months. After oral exposure of mice to As, we determined hearing levels by ABR and As levels in the inner ears by inductively coupled plasma mass spectrometry (ICP-MS). Before exposure to As, hearing levels in the control group (ctrl; n = 9) and exposure group (As; n = 8) were comparable (Fig. 3A). After exposure to As, hearing levels in the exposure group were significantly worse than those in the control group at 4 kHz (p = 0.0216), 12 kHz (p = 0.0167), 20 kHz (p = 0.0055) and 32 kHz (p = 0.0250) (Fig. 3B). Arsenic in the inner ears of the exposure group (As; n = 7) was detected at 0.3 µg/g of wet tissue weight and was significantly higher than that in the control group (ctrl; n = 5) (Fig. 4). We finally performed *ex vivo* exposure of the organ of Corti to As at 0.3 µg/mL in order to clarify the etiology of hearing loss by exposure to As at 0.3 µg/mL, which was the average level of As in inner ears of mice orally exposed to As. *Ex vivo* exposure to As at 0.3 µg/mL for 48 and 72 hours significantly decreased the number of neurofilament 200-positive auditory neurons but not the number of phalloidin-positive hair cells (Fig. 5).

## Discussion

Since a huge number of people worldwide are exposed to As via drinking water, it is important to evaluate the health risk of oral exposure to As. In this epidemiological study, it was shown by multivariate analysis with adjustments for age, sex, BMI and smoking that consumption of tube well water contaminated with As was significantly associated with hearing loss. As has been shown to accumulate in hair after chronic exposure to As in mice orally exposed to As via drinking water^13^. Correspondingly, our epidemiological data also showed significant correlations between As levels in hair and hearing loss at 4 kHz (r = 0.4816, p < 0.0001), 8 kHz (r = 0.5885, p < 0.0001) and 12 kHz (r = 0.6432, p < 0.0001) in humans (Figure S1). Thus, our epidemiological study showed for the first time that oral exposure to As via drinking water causes hearing loss in young people.

Our epidemiological study showed that frequencies of As-mediated hearing loss in people aged 12–29 years were 4, 8 and 12 kHz but not 1 kHz, while our experimental study showed that all frequencies were affected in young mice orally exposed to As. These results partially correspond to the results of our previous study showing that all frequencies of hearing were affected in a genetically engineered mouse model of age-related hearing loss and in mice orally exposed to toxic elements, while frequencies of hearing loss in control mice were limited to higher frequencies^14, 15^. Thus, it is possible that oral exposure to As accelerates the onset of age-related hearing loss in young people. On the other hand, exposure to environmental stresses including As, smoking and noise has been shown to cause oxidative stress^16–18^. A previous study showed that exposure to smoking affected high frequency of hearing in young people^11^. Also, exposure to broadband noise has been shown to affect high frequency of hearing in young mice at 1 month of age^19^. Therefore, it is possible that hearing levels at higher frequencies are more susceptible to oxidative stress caused by environmental factors than are those at lower frequencies.

The present experimental study demonstrated that oral exposure of young mice to As caused hearing loss with accumulation of As in inner ears. Thus, our combined study showed that oral exposure to As via drinking water causes hearing loss in young people and young mice. In the experimental study, mice were exposed to As at 22.5 mg/L for 2 months via drinking water. We used the dose for mice at the similar order as 10 mg/L and 100 mg/L of As that were based on the exposure doses for humans in a previous study^20^, although the dose used in our study (22.5 mg/L) is about 100-times higher than the maximum level (221 µg/L) of As detected in tube well water in this study. On the other hand, our epidemiological study showed that the duration of drinking tube well water was also associated with hearing loss at 4 kHz (r = 0.2685; p = 0.0182), 8 kHz (r = 0.4545; p < 0.0001) and 12 kHz (r = 0.4689; p < 0.0001) (Figure S2), suggesting that duration of oral exposure to As via drinking well water is associated with hearing loss in people aged 12–29 years. We therefore calculated a total exposure dose of As with consideration of the duration of drinking water. In our calculation with the exposure period of 7.94 years for humans (mean duration of drinking tube wells) and the exposure period of 2 months for mice, the difference between total exposure doses of As for mice (281 mg/kg) and humans (34 mg/kg) may be decreased to a difference of about 8 times. Further investigation of not only the accumulation levels of As but also hearing levels in other lines of haired mice in addition to the hairless mice used in this study after oral exposure to As at less than 22.5 mg/L is needed.

In our *ex vivo* study, exposure of the organ of Corti to As at 0.3 µg/mL, which was detected in inner ears of mice orally exposed to As *in vivo*, significantly decreased the number of auditory fibers and SGNs but not hair cells. Thus, our results indicate the possibility that 0.3 µg/g of As that accumulated in inner ears caused a decrease in the numbers of the auditory nerve fibers and SGNs *in vivo*. In previous studies, aquaporins (AQPs) including AQP9 were shown to mediate cellular uptake of As in mammalian cells^21^. AQP9 was shown to be expressed in SGNs but not hair cells in the organ of Corti^22^. Therefore, it is possible that As accumulated in SGNs via AQPs caused a decrease in the numbers of auditory nerve fibers and SGNs. Previous studies also showed that As-mediated neurotoxicity was involved in apoptosis and demyelination of peripheral neurons including dorsal root ganglion (DRG) neurons^23, 24^. As-mediated neurotoxicity was also shown to be involved in increased levels of oxidative stress markers including heme oxiygenase 1 (HO-1) and decreased levels of procaspases 3 and 12 in DRG explants^25^. Further study is needed to determine whether *in vivo* exposure to As induces oxidative stress, apoptosis and demyelination in auditory neurons and to elucidate the molecular mechanism by which hair cells were not affected by exposure to As in this study.

In conclusion, our combined study suggests that exposure to As via drinking water causes hearing loss in young people and young mice. Hearing loss in young people has a negative impact on the quality of life. Therefore, further study is needed to determine the correlation between hearing loss and oral exposure to As via drinking water in order to prevent As-mediated hearing loss in humans.

## Materials and Methods

### Epidemiological study

We performed a survey using self-administered questionnaire regarding age, smoking, sex, weight and height for 77 healthy subjects aged 12–29 years in Bangladesh. Informed consent in written form was obtained from all of the participants in the study. Subjects in the control group and subjects in the exposure group were drinking tap water and tube well water contaminated with As, respectively. Subjects with a drinking habit or a habit of using earphones to listen a music did not participate in this study. In addition, another ethnic group or race was not included as subjects in this study. In brief, BMI was calculated as weight (kg) divided by the square of height (m^2^). In this study, subjects were defined as being underweight if their BMI was less than 18.5 kg/m^2^, being of normal weight if BMI was 18.5–24.9 kg/m^2^ and being overweight if BMI was higher than 25 kg/m^2^ as set by the WHO^26^. We measured hearing thresholds at 1, 4, 8 and 12 kHz in the subjects by pure tone audiometry (PTA)^10, 11, 27^. For each frequency, the sound pressure level was increased from 0 dB to 90 dB in 5-dB steps until the subject showed a response. The epidemiological study was approved by Nagoya University International Bioethics Committee following the regulations of the Japanese government (approval number 2013-0070) and the Faculty of Biological Science, University of Dhaka (Ref. no. 5509/Bio.Sc).

### Experimental study

Hairless mice having a C57BL/6 J mice background were purchased from Hoshino Laboratory Animal, Inc. The mice were maintained under specific pathogen-free (SPF) conditions at a fixed temperature (23 ± 2°C) and a 12-h light/dark cycle. Sodium arsenite (NaAsO_2_) purchased from Sigma-Aldrich was dissolved in distilled water. We newly prepared drinking water containing As and changed the drinking water every week. We gave distilled water to the non-exposure group. We started the exposure experiment at 1 month of age. ABR measurements (AD Instruments Pty. Ltd.) were performed as described previously^14, 15, 28^. Tone burst stimuli were measured 5 dB-stepwise from 0 dB SPL to 90 dB SPL. The threshold was judged by the appearance of the lowest level of I-IV waves of ABR. Data are presented as means ± SD. The experimental study was approved by the Institutional Animal Care and Use Committee in Nagoya University (approval number: 28251) and followed the Japanese Government Regulations for Animal Experiments.

### Determination of As levels in biological samples

We collected biological samples (toenails, hair and urine) and performed measurements by ICP-MS (Agilent 7500cx) as described previously^14, 15, 27^. In brief, samples were placed in 15 ml tubes and ashed overnight at room temperature with 3 ml HNO_3_. Before ashing, nails were washed with detergent water and kept at room temperature overnight. After heating the samples at 80°C for 3 hours, 1–1.5 ml (0.5 ml for mice) H_2_O_2_ was added and the samples were immediately incubated at 80°C for 3 hours. After cooling, the samples were diluted by Milli-Q water. For urinary samples, a 15 ml tube was filled with 4 ml of a urine sample followed by addition of 1 ml HNO_3_, and the tube was left overnight. The samples were incubated at 80°C for 24–72 hours until the solution became clear. After centrifuging for 1 min, the samples were adjusted with Milli-Q water up to 5 ml, and the As levels were measured by ICP-MS. Urinary As levels were normalized for dilution by specific gravity (SG) adjustment using the following formula^27^:

$${\rm{S}}{\rm{G}}{\textstyle \text{-}}{\rm{c}}{\rm{o}}{\rm{r}}{\rm{r}}{\rm{e}}{\rm{c}}{\rm{t}}{\rm{e}}{\rm{d}}\,{\rm{c}}{\rm{o}}{\rm{n}}{\rm{c}}{\rm{e}}{\rm{n}}{\rm{t}}{\rm{r}}{\rm{a}}{\rm{t}}{\rm{i}}{\rm{o}}{\rm{n}}={\rm{r}}{\rm{a}}{\rm{w}}\,{\rm{h}}{\rm{o}}{\rm{r}}{\rm{m}}{\rm{o}}{\rm{n}}{\rm{e}}\,{\rm{c}}{\rm{e}}{\rm{n}}{\rm{t}}{\rm{r}}{\rm{a}}{\rm{t}}{\rm{i}}{\rm{o}}{\rm{n}}\times {[({\rm{S}}{\rm{G}}}_{{\rm{t}}{\rm{a}}{\rm{r}}{\rm{g}}{\rm{e}}{\rm{t}}}-1{.0)/({\rm{S}}{\rm{G}}}_{{\rm{s}}{\rm{a}}{\rm{m}}{\rm{p}}{\rm{l}}{\rm{e}}}-1.0)]$$

where SG_target_ is a population mean SG. In this study, the target SG used was 1.012 for subjects. The levels of As in inner ears were determined by the same protocol as that described above.

### *Ex vivo* culture of the organ of Corti

We partially followed the previous method of *ex vivo* culture of the organ of Corti^29^. In brief, after dissection of the organs of Corti from C57BL6/J mice at postnatal days 4–6, the cochleas were placed in HBSS buffer. The cochlears were dissected and the organs of Corti containing IHC, OHC, auditory nerve fibers (ANFs) and SGNs were placed in a 6-well dish. D-MEM (low glucose) medium containing 10% FBS and 10 µg/mL of ampicillin was added to the dish. A 0.3 mg/mL stock solution of As was diluted in the culture medium to a final concentration of 0.3 µg/mL. The organ of Corti was placed in an incubator (37°C, 5% CO_2_). On the next day (day 1), we removed the medium and added a fresh medium for the control group and exposure group (0.3 µg/mL of As). On day 2 and day 3, the medium was removed and the organ of Corti was washed three times with 0.1 M PBS. Specimens were fixed with 10% formalin for 1 hour and rinsed with PBS. To detect SGNs and auditory fibers, specimens were immunolabeled with a solution containing 1 µL of rabbit anti-neurofilament 200 kD (Sigma, N4142) primary antibody, 40 µL of 10% Triton X-100, 6 µL of normal goat serum and 153 µL of PBS overnight at 4°C. On the second day, specimens were washed with PBS and immersed in a solution containing 1 µL of a secondary antibody, 20 µL of 10% Triton X-100, 6 µL of normal goat serum and 173 µL of PBS for 2 hours at room temperature. To evaluate hair cells, we labeled specimens with fluorescein-phalloidin (Wako, 068-06261). The specimens were immersed in a solution containing 1 µL of fluorescein-pholloidin (6.6 µM stock solution) and 199 µL of PBS. After washing with PBS, the specimens were mounted on glass sides, covered with antifade reagent, and observed with Super-resolution/confocal Microscopy (Carl Zeiss Microscopy LSM880-ELYRA PS.1). Hair cells were counted in the width (100 µm) of the field of view of the of microscope at 40X. The ANFs (length > 50 µm) and SGNs were counted in the field (100 µm × 100 µm) of view of the microscope at 40X. Ten specimens were examined for each group.

### Statistical analysis

All statistical analyses were performed by JMP Pro (version 11.0.0; SAS Institute Inc., Cary, NC, USA) as described previously^27^. Since the Shapiro-Wilks test showed that As levels in biological samples and hearing thresholds for PTA and ABR of all frequencies were non-normal distributions, the Mann-Whitney *U* test to evaluate statistical differences of As levels and hearing levels between two groups. For multivariate analysis, we performed binary logistic regression analysis with adjustments for age^8^, sex^9^, BMI^12^ and smoking^10, 11^ as confounding factors. In this study, values of p < 0.05 were considered statistically significant.

## Electronic supplementary material

Supplementary Information
