# Supplementary material for: Oral exposure to arsenic causes hearing loss in young people aged 12–29 years and in young mice
Source: Sci Rep. 2017 Jul 28;7:6844. doi: 10.1038/s41598-017-06096-0 (PMC5533757; doi:10.1038/s41598-017-06096-0)
Supplement: Supplementary file 1 — Supplementary Information [file 41598_2017_6096_MOESM1_ESM.pdf]

Oral exposure to arsenic causes hearing loss in young people aged 12-29 years and in young mice

Xiang Li[1], Nobutaka Ohgami[1,2,3], Yasuhiro Omata[1], Ichiro Yajima[1,3], Machiko Iida[1], Reina Oshino[1,3], Shoko Ohnuma[3], Nazmul Ahsan[3,4], Anwarul Azim Akhand[3,4] and Masashi Kato[1,3,*]

Author Affiliation:

[1]Department of Occupational and Environmental Health, Nagoya University Graduate School of Medicine, Nagoya, Japan.

[2]Nutritional Health Science Research Center, Chubu University, 1200 Matsumoto, Kasugai, Aichi 487-8501, Japan.

[3]Voluntary Body for International Health Care in Universities, Nagoya, Japan.

[4]Department of Genetic Engineering and Biotechnology, University of Dhaka, Dhaka-1000, Bangladesh.

[*]Correspondence:

Masashi Kato M.D., Ph.D.

Department of Occupational and Environmental Health,

Nagoya University Graduate School of Medicine

Address: 65 Tsurumai-cho, Showa-ku, Nagoya, Aichi 466-8550, Japan.

Phone: +81-52-744-2122. Fax: +81-52-744-2124.

E-mail: katomasa@med.nagoya-u.ac.jp

Supplementary information

Figure legend

Figure S1. Correlations of hearing thresholds in people aged 12-29 years with As levels in hair. Correlations of hearing levels (dB) at 1 kHz (A), 4 kHz (B), 8 kHz (C) and 12 kHz (D) with As levels in hair (μg/g) were determined by Spearman correlation coefficients.

Figure S2. Correlations of hearing thresholds in people aged 12-29 years with duration of drinking tube well water. Correlations of hearing levels (dB) at 1 kHz (A), 4 kHz (B), 8 kHz (C) and 12 kHz (D) with duration of drinking tube well water (years) were determined by Spearman correlation coefficients.

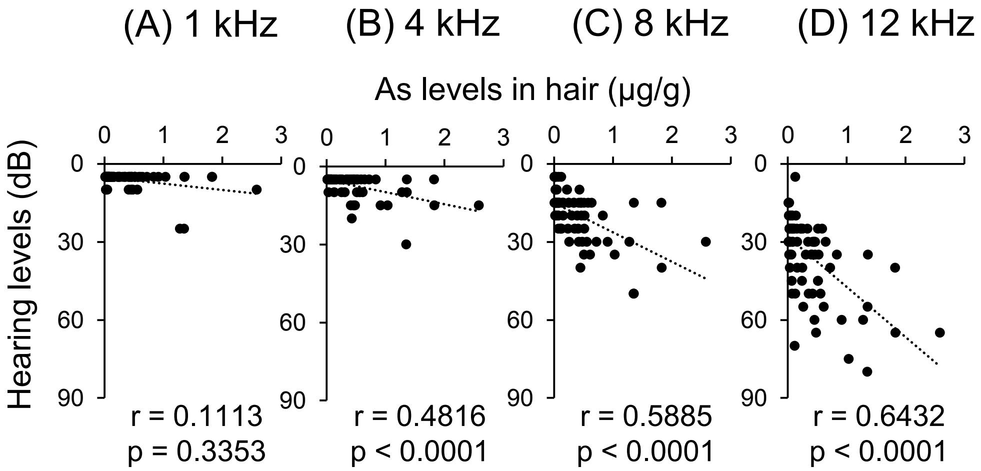

Li et al. Figure S1

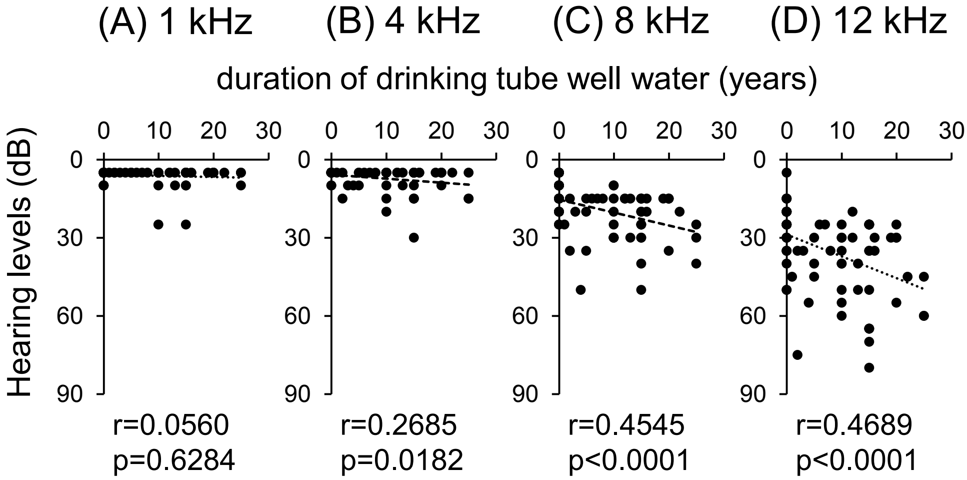

Li et al. Figure S2
